# Learning about Emotions in Illness: Integrating Psychotherapeutic Teaching into Medical Education

**DOI:** 10.1192/pb.bp.114.048785

**Published:** 2015-06

**Authors:** Paramabandhu Groves

**Figure:**
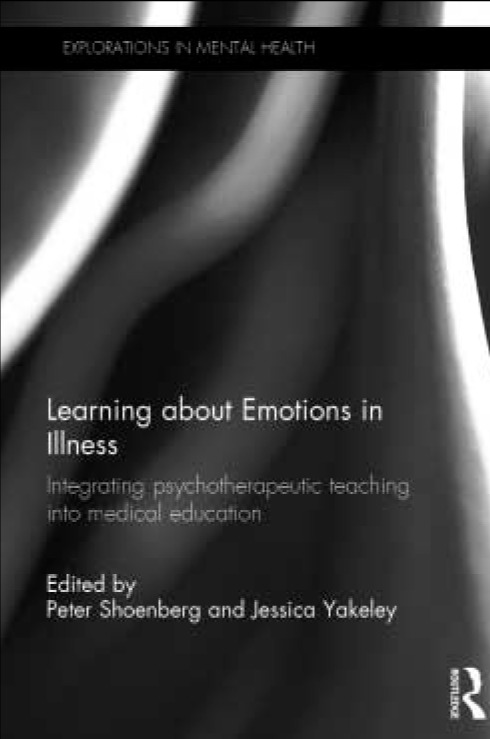


Reading *Learning about Emotions in Illness*, I found myself reflecting on my emotional response to the book – I was moved, and surprised at being moved. Partly, it reminded me of my own time as a medical student participating in the student psychotherapy scheme, which gave me my first opportunity of being useful as a trainee doctor, as opposed to being someone in the way. The supervision group was a wonderful and constant haven in which to reflect within the busyness and ever-changing landscape of medical training. Partly, I was simply moved by some of the accounts of people who as students had participated in either of the schemes described in the book, as they grappled with their own and their patients’ emotional responses, especially to physical illness.

The book describes two approaches aimed at helping students learn how psychotherapeutic understanding can help them with their patients: the student psychotherapy scheme and student Balint groups. There are accounts of the scheme both from its supervisors and from participants, and there is also a chapter on research into the two schemes. The University College London student psychotherapy scheme has a long pedigree, having run for over 50 years and surviving various organisational changes. It has spawned other schemes such as in Bristol and Heidelberg. The scheme allows medical students to take on a patient for psychotherapy for a period of about a year. At its inception, allowing untrained students to practise psychotherapy was an audacious move. However, patients are carefully selected and the process is well supervised, and studies seem to indicate that patients have a good outcome. For students, the scheme often leaves an indelible mark, with a number of people citing it as a highlight of their medical training.

By its nature the psychotherapy scheme can only take on a limited number of students, and numbers wanting to participate outstrip the available places. Modified student Balint groups were introduced at University College London as an alternative. These meet in small groups for a period of 11 weeks and are used to reflect on students’ emotional responses to patients they have seen, to help foster, in Balint’s terms, a patient-centred rather than an illness-centred approach.

Participants in the scheme seem more likely to become psychiatrists – a point to be noted given the recruitment shortage. However, the real value of these schemes is in helping to develop doctors who can tolerate difficult emotions that arise in patient–doctor interactions and to be alive to the often unspoken emotions that our patients communicate. In other words, regardless of specialty, to make better doctors.

